# Cardiac Cell Patterning on Customized Microelectrode Arrays for Electrophysiological Recordings

**DOI:** 10.3390/mi12111351

**Published:** 2021-10-31

**Authors:** Jiaying Ji, Xiang Ren, Pinar Zorlutuna

**Affiliations:** Department of Aerospace and Mechanical Engineering, University of Notre Dame, Notre Dame, IN 46556, USA; jji2@nd.edu (J.J.); xren2@nd.edu (X.R.)

**Keywords:** microelectrode array, cardiomyocytes, human pluripotent stem cells, micro-patterning

## Abstract

Cardiomyocytes (CMs) and fibroblast cells are two essential elements for cardiac tissue structure and function. The interactions between them can alter cardiac electrophysiology and thus contribute to cardiac diseases, such as arrhythmogenesis. One possible explanation is that fibroblasts can directly affect cardiac electrophysiology through electrical coupling with CMs. Therefore, detecting the electrical activities in the CM-fibroblast network is vital for understanding the coupling dynamics among them. Current commercialized platforms for studying cardiac electrophysiology utilize planar microelectrode arrays (MEAs) to record the extracellular field potential (FP) in real-time, but the prearranged electrode configuration highly limits the measurement capabilities at specific locations. Here, we report a custom-designed MEA device with a novel micropatterning method to construct a controlled network of neonatal rat CMs (rCMs) and fibroblast connections for monitoring the electrical activity of rCM-fibroblast co-cultures in a spatially controlled fashion. For the micropatterning of the co-culture, surface topographical features and mobile blockers were used to control the initial attachment locations of a mixture of rCMs and fibroblasts, to form separate beating rCM-fibroblast clusters while leaving empty space for fibroblast growth to connect these clusters. Once the blockers are removed, the proliferating fibroblasts connect and couple the separate beating clusters. Using this method, electrical activity of both rCMs and human-induced-pluripotent-stem-cell-derived cardiomyocytes (iCMs) was examined. The coupling dynamics were studied through the extracellular FP and impedance profile recorded from the MEA device, indicating that the fibroblast bridge provided an RC-type coupling of physically separate rCM-containing clusters and enabled synchronization of these clusters.

## 1. Introduction

While cardiomyocytes (CMs) are known as the vital cell type for heart contraction, two-thirds of heart cells are non-cardiomyocytes, among which cardiac fibroblasts constitute the largest fraction. The interaction between the CMs and fibroblasts can alter cardiac electrophysiology and thus contribute to arrhythmogenesis. The underlying mechanisms, however, remain unknown. Traditionally, fibroblasts are considered as the electrical barriers against conduction by producing insulating collagenous septa [1]. There is increasingly more research indicating that fibroblasts can also directly affect the cardiac electrophysiology through electrical coupling with CMs [2]. Such electrical coupling between CMs and fibroblasts is achieved via action potential propagation, which is caused by ion fluxes through their heterocellular gap junctions [3]. One technology capable of detecting the electrical activity in the CM-fibroblast network is the microelectrode array (MEA) platform. Conventional electrophysiological monitoring methods, including patch-clamp, brightfield video-based and fluorescent dye-based assessment, are invasive and require further investigation in the relationship between contraction and electrical activity [4,5,6]. However, MEAs that record the extracellular field potential (FP) of the attached cells on the electrodes [7,8] have the advantages of being non-invasive, high throughput and compatible with other detection methodologies, such as atomic force microscopy (AFM) or ultrafast imaging [9,10]. The MEA system was first proposed by Thomas in 1972 as a miniature platform to monitor the electrical activity of contracting heart cells in vitro [7]. After decades of development, MEAs have become a promising and commercialized platform for studying cardiac electrical activity, such as investigating the synchronization of cultured cardiac cells taken from different origins [11], evaluating the maturation of anisotropic human-induced-pluripotent-stem-cell-derived cardiomyocyte (iCMs) or measuring the extracellular FP changes of a single cardiac cell [12]. However, the pre-arranged electrode design layout of commercial MEA systems highly limits the measurement capabilities at specific locations. Therefore, fabrication methods to create custom-designed MEAs with electrode arrangements that could match the cell-level organization would be valuable.

To study the coupling dynamics in the CM-fibroblast networks using a customized MEA device, here we leveraged cell micropatterning techniques to control the cardiac cells’ localization and the interaction among them. Cell micropatterning methods enable control over cell-cluster shape, size, or organization in vitro by using microfabrication techniques including photolithography, microprinting and electrospinning, as well as various materials such as polymers or metals. Current micropatterning methods in cardiac tissue engineering are mainly used for constructing anisotropic cardiac tissues by changing the culture substrate physically or chemically, such as employing microgrooves [13], 3D bioprinted hydrogels [14], topographical features or attachment of proteins [15]. However, there is limited research on constructing an organized CM-fibroblast network to investigate the coupling between them. In a recent study, Kaneko et al. [16] examined the beating synchronization of two CMs connected by a single fibroblast using micropipette technology. However, this single-cell-based approach has weak coupling strength and may damage cells during the procedure. Here, we combine the surface topographic features with mobile blockers, providing a tunable micropatterning method to construct a controlled network of CM and fibroblast connections for studying the coupling dynamics among them. 

Recently, micropatterning has also been utilized to enhance the maturation of iCMs. iCMs provide a physiologically relevant cell model to eliminate interspecies differences and have great potential in therapeutic benefits in myocardial regeneration. However, compared to the native CMs, iCMs still display some immature qualities, such as poor sarcomeric organization [17] or lower upstroke and conduction velocities [18]. Micropatterning techniques provide topographical cues that can align the iCMs, increase the anisotropic contractility and better mimic the native environment. Combining the micropatterning method with a customized MEA device, we are able to detect the electrophysiological properties of patterned iCMs to evaluate the maturation or further study the synchronization among iCMs.

In this paper, we present a micropatterning method on a custom-designed MEA device that allows the long-term monitoring of the electrical activity of a CM-fibroblast network in a spatially controlled fashion. Using surface topographic features and mobile blockers, the localization of cardiac cells was defined in the permissive areas as separated clusters, and the interconnection between them was formed by fibroblast bridges. This customized MEA device can also be used to assess the electrophysiological properties of patterned iCMs. 

## 2. Materials and Methods

### 2.1. MEA Device Fabrication 

**Figure 1:**
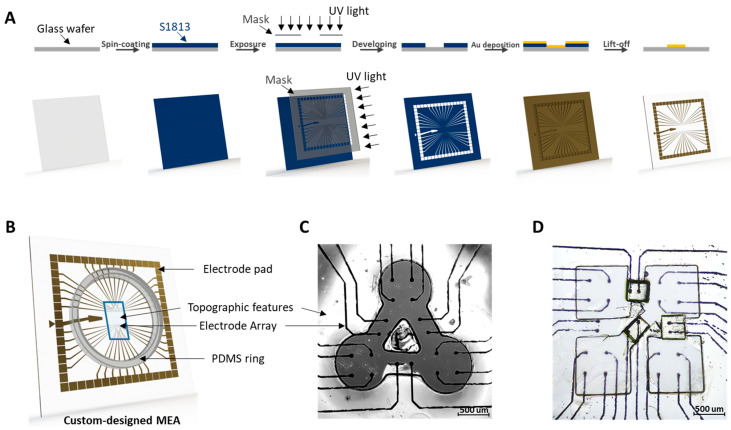
(**A**) Fabrication process of the MEA substrate. (**B**) 3D schematic diagram of custom-designed MEA device. (**C**,**D**) Bright field image of three- and four-cluster MEA with corresponding surface topographic features. Scale bar = 500 µm.

The custom-designed MEAs were fabricated by photolithography and metal evaporation (Figure 1A). Glass wafers, 10 cm in diameter, were coated with S1813 positive photoresist (Shipley 1813, Kayaku Advanced Materials, Inc., Westborough, MA, USA) at 2000 rpm and baked at 95 °C for 5 min. After baking, the wafers were exposed to UV light through a negative photomask of the desired pattern and then immersed in 351 developer (Kayaku Advanced Materials, Inc., Westborough, MA, USA) to remove the exposed photoresist. Chrome (Cr) and gold (Au) were successively deposited on the wafers at thicknesses of 20 nm and 100 nm, respectively, by the electron beam vacuum deposition system (FC-1800, AIRCO Temescal). The wafers with deposited metal were then immersed in acetone to dissolve the excess photoresist while removing the excess Cr and Au, which left a gold image of the custom-designed MEA. Then the wafers were coated with a layer of S1813 at 800 rpm to protect the gold electrode patterns during wafer cutting with a dicing saw (Model DAD 3240, DISCO). The dimensions of the cut-out square from the wafer were selected so that the customized MEAs are compatible with the commercial MEA-2100 system (Multichannel Systems) that we used for electrophysiological measurements. For the impedance measurements, we used a potentiostat (Gamry^®^ Reference 600, Gamry Instruments, Warminster, PA, USA). A polydimethylsiloxane (PDMS) ring fabricated by soft-lithography was then bonded with MEA substrate using a plasma cleaner (Harrick-Plasma PDC-001, Ithaca, NY, USA) to create a culture chamber for the long-term cell activity recording experiments. The details in the soft-lithography fabrication that we used for creating the patterns and the culture chamber are explained in the sections below. 

### 2.2. Surface Topographic Feature and Blocker Fabrication

The PDMS features fabricated by soft-lithography were bonded on the surface of the MEA substrates to define the cell patterns and the bridges between clusters of cardiac cells. The clusters were patterned into circles with a diameter of 800 µm connected by the bridges with a dimension of 500 µm × 300 µm. 

The SU-8 3050 photoresist (Kayaku Advanced Materials, Inc., Westborough, MA, USA) was spin-coated on a silicon wafer at 800 rpm for 30 s to achieve a thickness of 140 µm. After baking at 65 °C for 15 min and 95 °C for 60 min, the wafer was exposed to UV light at a dose of 250 mJ/cm^2^ through a negative photomask of the desired pattern. Then the wafer was baked again at 65 °C for 10 min and 95 °C for 30 min to further cure the SU-8. The wafer was immersed in SU-8 developer (Kayaku Advanced Materials, Inc., Westborough, MA, USA) to remove the uncured photoresist and cleaned with isopropanol and DI water. Tridecafluoro-1,1,2,2-tetrahydrooctyl-1-trichlorosilane (TFOCS, Fisher Scientific, Waltham, MA, USA) was vacuum-coated on the surface of the silicon wafer mold for the easy release of PDMS. Then standard PDMS replica molding was conducted to fabricate PDMS features. PDMS pre-polymer (SYLGARD^®^ 184 silicone elastomer, Dow Corning, Midland, MI, USA) and curing agent (SYLGARD^®^ 184 silicone elastomer curing agent, Dow Corning, Midland, MI, USA) were mixed at a weight ratio of 10:1 and then poured onto the SU-8 mold. After degassing and baking, the PDMS features were manually removed from the mold and bonded with MEA substrate using the plasma cleaner to obtain the surface topographic features. The SU-8 blockers with a dimension of 300 µm × 320 µm were fabricated by the same process of photolithography. The width of blockers (320 µm) is slightly wider than the bridge width (300 µm) in order to blockade the bridge area. 

### 2.3. Cell Patterning and Culture

Rat CMs were isolated from two-day-old Sprague Dawley rat hearts following a previously established protocol [19] in compliance with the IACUC guidelines and under an approved protocol from the University of Notre Dame. The differentiation induction of iCMs followed our previously established protocols [20]. Briefly, DiPS 1016 SevA human induced pluripotent stem cells (hiPSCs) derived from human skin fibroblasts (Passage number 40–50) were seeded on a Geltrex-coated (1% Invitrogen, Carlsbad, CA, USA) tissue culture flask using mTeSR (StemCell Technologies, Vancouver, BC, Canada) supplemented with 1% penicillin (VWR, Radnor, PA, USA). At 80% confluence, hiPSCs were detached and reseeded into the culture cell-plate and incubated in mTeSR1 media supplemented with Rho-associated, coiled-coil containing protein kinase (ROCK) inhibitor (5 µM, StemCell Technologies, Vancouver, BC, Canada). The media were changed every day until the cells reached 95% confluence. Then, hiPSCs were treated with RPMI Medium 1640 (Life Technologies, Carlsbad, CA, USA) supplemented with B27 without insulin (2%, Invitrogen, Carlsbad, CA, USA), beta-mercaptoethanol (final concentration of 0.1 mM, Promega, Madison, WI, USA) and penicillin (1%) (CM (-)) with the addition of Wnt activator, CHIR99021 (CHIR) (12 µM, Stemgent, Cambridge, MA, USA). Twenty-four hours later, media were changed to CM (-) without any CHIR. On day 4, iCMs were treated with CM (-) media supplemented with the Wnt inhibitor IWP-4 (5 µM, Stemgent, Cambridge, MA, USA), media were changed back to CM (-) two days later. On day 9, iCMs were cultured with RPMI Medium 1640 supplemented with B27 (2%, Invitrogen, Carlsbad, CA, USA), beta-mercaptoethanol (final concentration of 0.1 mM) and penicillin (1%) (CM (+)). After day 9, the media were changed every 3 days, and beating was observed routinely by day 21 of differentiation. Before cell seeding, the blockers were manually placed at the central location of bridges and then treated with fibronectin to allow cell adhesion. Both cell types were seeded at a density of 1 million cells/mL. Once cardiomyocyte clusters started to beat, the blockers were removed to allow the proliferation of fibroblasts. The culture of rCMs was maintained under standard cell culture conditions in Dulbecco’s modified Eagle medium (DMEM) (Hyclone, Logan, UT, USA) supplemented with fetal bovine serum (FBS) (10%, Hyclone, Logan, UT, USA) and penicillin (1%, Life Technologies, Carlsbad, CA, USA). iCMs were cultured in RPMI Medium 1640 (Life Technologies, Carlsbad, CA, USA) supplemented with B27 (2%, Invitrogen, Carlsbad, CA, USA), beta-mercaptoethanol (3.4 × 10^−4^%, Promega, Madison, WI, USA) and penicillin (1%) (CM (+)). The media were changed every day. 

### 2.4. Immunofluorescence Staining

Immunofluorescence staining was employed to visualize the rCM and fibroblast patterning on the MEA substrate. Cells were fixed with 4% paraformaldehyde (Electron Microscopy Sciences, Hatfield, PA, USA) for 20 min at room temperature, followed by washing with phosphate-buffered saline (PBS) 3 times. Cells were then permeabilized in Triton X-100 (0.1%, Sigma-Aldrich, St. Louis, MO, USA) for 30 min and then washed 5 times with PBS. Cells were blocked by goat serum (5%, Sigma-Aldrich, St. Louis, MO, USA) for 1 h, and incubated with Vimentin (Abcam, Cambridge, UK), or Troponin T (Abcam, Cambridge, UK) primary antibody diluted (1:150) in goat serum at 4 °C overnight. Then, cells were washed 5 times with PBS and then incubated with Alexa Fluor 594 (Life Technologies, Carlsbad, CA, USA) and Alexa Fluor 488 (Life Technologies, Carlsbad, CA, USA) secondary antibody diluted (1:200) in goat serum at 4 °C for 4 h. After incubation, cells were washed with PBS again and incubated with DAPI (1:1000 DAPI:PBS, Sigma Aldrich, St. Louis, MO, USA) and then washed 5 times. Imaging was performed using a fluorescence microscope (Axio Observer.Z1, Zeiss, Germany, Hamatsu C11440 digital camera, Shizuoka, Japan).

### 2.5. Ca^2+^ Indicator Loading and Video Analysis

Rat CMs were loaded with Fluo-4 acetoxymethyl ester (Molecular Probes, Eugene, OR, USA), which exhibits an increase in fluorescence intensity upon binding to Ca^2+^. The Ca^2+^ indicator was diluted with an equal volume of 20% (*w*/*v*) Pluronic in DMSO (Sigma-Aldrich, St. Louis, MO, USA) to a final Pluronic concentration of 0.02% in Tyrode’s solution. Cells were cultured in the solution at 37 °C for 30 min and washed with an indicator-free media after loading. The Ca^2+^ fluorescent measurements were performed using a microscope (Axio Observer.Z1, Zeiss, Germany, Hamatsu C11440 digital camera, Shizuoka, Japan). The Ca^2+^ fluxes were analyzed based on the change in gray level between two successive frames in the fluorescent videos. 

### 2.6. Electrical Data Analysis

FP was recorded by the MEA-2100 system (Multichannel Systems, Reutlingen, Germany) with a sampling rate of 1 kHz, and the data were analyzed by MATLAB. 

Three primary electrophysiologic characteristics of iCMs were derived from FP data: spike amplitude, beat period, and field potential duration (FPD). At least 40 min beating prior to the asynchronous contractions was analyzed and averaged. The spike amplitude was defined as the potential difference between the positive and negative peak of the initial rapid spike. The beat period was defined as the time period between two successive rapid spikes. The FPD was defined as the time difference between the initial rapid spike and the end of the subsequent spike. The endpoint upon which the second spike returned to the baseline was identified manually. To diminish the effect of beat rate on FPD, the Fridericia formula was employed to correct FPD (cFPD): $cFPD=FPD\;/beat\;period^{\frac{1}{3}}.\;$

## 3. Results and Discussion

### 3.1. MEA Sensor Device and Topographic Feature Fabrication

We designed a customized MEA device (Figure 1B) that matches the micropattern of three interconnected clusters where electrodes are positioned explicitly at each cluster and bridge. Electrodes located at clusters can be used to detect the FP from contracting rCMs, while the electrodes located at the bridges can be used to obtain propagation signals (Figure 1C). Electrode pads around the MEA are designed to connect the detection system with 60 μm-diameter electrodes by 400 µm lines, which shrink to 20 µm near the electrode. The lines inside the culture chamber are coated with PDMS as an insulation layer to avoid unexpected electrical signals from attached cells. By the standard lithography and metal deposition process, this methodology of customizing MEAs can also be applied in more complex patterns, as shown in Figure 1D. 

### 3.2. Cell Patterning with Surface Topographic Features

**Figure 2:**
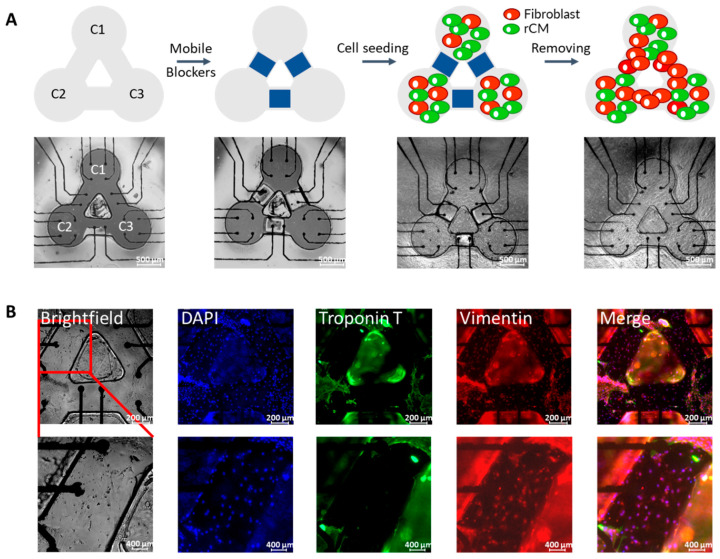
(**A**) Schematic of rCM patterning on three-node custom-designed MEA device. Scale bar = 500 µm. (**B**) Immunostaining results of three separate rCM clusters connected by fibroblast. The scale bars are 200 µm for the upper row and 400 µm for the lower row.

To create a controlled network of rCM and fibroblast connections on the customized MEA, we used a cell patterning approach (Figure 2A). The PDMS-based topographic features were bonded with the MEA substrate to define areas that were permissive to cell binding by allowing fibronectin to get adsorbed in these sections of the pattern while mobile blockers were used to obstruct initial cell attachment to the bridges between these cell permissive areas. Then, freshly isolated neonatal rat cardiac cells, a mixture of rCMs and fibroblasts, were seeded into the surface of the MEA device. Mammalian CMs lose their regeneration ability shortly after birth, while the fibroblasts can proliferate under proper culture conditions. Therefore, after the blockers were removed, the fibroblasts in the mixture of cardiac cells would proliferate and occupy the bridges that were initially obstructed by blockers, hence connecting the adjacent beating rCM clusters. Immunofluorescence staining results were used to observe cell distribution and fibroblast connection on the MEA device (Figure 2B). The nuclei and Troponin T (blue and green, respectively) can be seen clearly in the cluster areas, but there are no Troponin T signals in the bridges, verifying that blockers successfully obstructed the initial cell attachment to the bridges. However, the appearance of vimentin (red), a fibroblast marker, in the bridges indicates fibroblast growth. The immunostaining results verify that our cell micropatterning method is useful for defining the controlled connections among the rCMs and fibroblasts. In this paper, the three blockers were all removed together to obtain an interconnected pattern, but we can also achieve other patterns by adjusting the number of removed blockers or by controlling the removal sequence of the blockers. 

### 3.3. Extracellular Recording of the Synchronized rCM-Fibroblast Network

**Figure 3:**
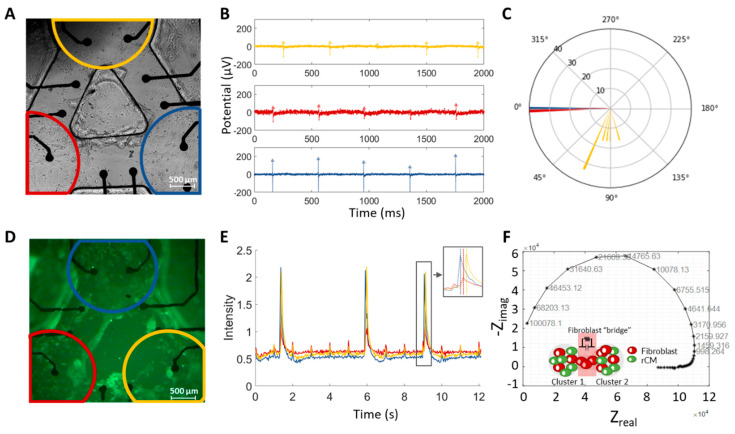
(**A**) Brightfield image of three rCM clusters on the MEA device. (**B**) The representative rCM electrical signals recorded by custom-designed MEA. (**C**) Polar histogram of the phase difference shown in (**B**). (**D**) Ca^2+^ fluorescent image of three rCM clusters. (**E**) Intensity plot of the phase difference of Ca^2+^ fluxes shown in fluorescent video. (**F**) Nyquist plot of fibroblast bridge which is represented as an RC filter with resistance of 200 kΩ and capacitance of 120 nF.

The extracellular potential changes of the synchronized rCM-fibroblast network were monitored with the custom-designed MEA device. Figure 3A shows a brightfield image of how cells were patterned on the MEA device. When the synchronized contraction of the three-patterned rCM clusters was observed from the microscope (data are not shown), the MEA was placed in the detection system for recording. The transmembrane potentials propagating through cardiac cells polarize the MEA electrodes and cause the electrode potential changes, which are recorded as the FP.

Figure 3B shows representative synchronized electrical signals of the three-cluster rCM-fibroblast network. The three clusters are presented in blue, red, and yellow. The peak values of the electrical signals are obvious and regular, and the spike amplitude ranges from 100 μV to 400 μV, which is similar to previous studies on commercial MEA [9,21]. By comparing the waveforms of the three clusters, the steady phase differences between the coupled clusters can be observed. We used cluster 1 (blue) as the reference cluster to measure the phase difference of clusters 2 and 3 (red and yellow). The polar histogram of Figure 3B is shown in Figure 3C regarding the spontaneous beating as a periodic function. The phase differences are measured in the angular unit, and the height of the histogram represents the percentage of the coupled period during which the phase difference is at a specific angle. The phase differences of clusters 2 and 3 compared to cluster 1 are around 5° and 67°, respectively. Such phase differences can also be observed from fluorescent videos simultaneously owing to the transparent substrate of the MEA device. We recorded Ca^2+^ fluorescent videos with Fluo-4 to visualize the Ca^2+^ flux (Figure 3D, Supplementary Movie 1). The video shows that Ca^2+^ (green) was diffused among the rCM clusters synchronously and consecutively. The results of quantified Ca^2+^ fluxes analyzed by pixel intensities of the fluorescent signals in the video are shown in Figure 3E. The peaks with intensity larger than 1 represent the three beatings in the video, while the peaks with intensity around 0.7 represent the background glows caused by the light reflection of topographical features. The magnified peaks indicate stable phase differences among the three clusters (right upper corner in Figure 3E). 

In order to explore the coupling between rCMs and fibroblasts, we measured the impedance of the fibroblast bridge in the customized impedance MEA device. The fabrication process of the impedance sensors was the same as shown in Figure 1D. The Nyquist plot (Figure 3F) shows that the fibroblast bridge can be presented as an RC filter with a resistance of 200 kΩ and capacitance of 120 nF, providing an RC-type coupling among synchronized rCM clusters.

rCMs can spontaneously generate electrical signals and beat at the same frequency when coupled. This coupling is achieved by propagating electrical signals through the gap junctions of adjacent cells. Here, after fibroblasts grew in the bridges and connected the three rCM clusters, these three clusters would initiate the calcium exchange through the gap junctions in the fibroblast bridges and then beat at the same pace. The time lag in transporting electrical signals via the fibroblast bridges led to the beating phase differences between the coupled rCM clusters, as shown in both electrical and optical results. In our previous paper [22], we verified that the phase difference between clusters is modulated by the fibroblast length and synchronized frequency. Here, although we kept the length of the fibroblast bridges constant, the inherent variation in the initial and final synchronized frequencies of CMs from different biological replicates can result in variation in the phase differences (Figure 3A,D).

The above results indicate that the custom-designed MEA device can be used for studying the coupling dynamics in a controlled network of rCM and fibroblast connections. The electrical data analyzed from MEA and quantized Ca^2+^ fluxes analyzed from optical videos reveal that the proliferated fibroblast bridges provide an RC-type coupling and induce stable phase differences among coupled rCM clusters.

### 3.4. Extracellular Recording of iCMs

**Figure 4:**
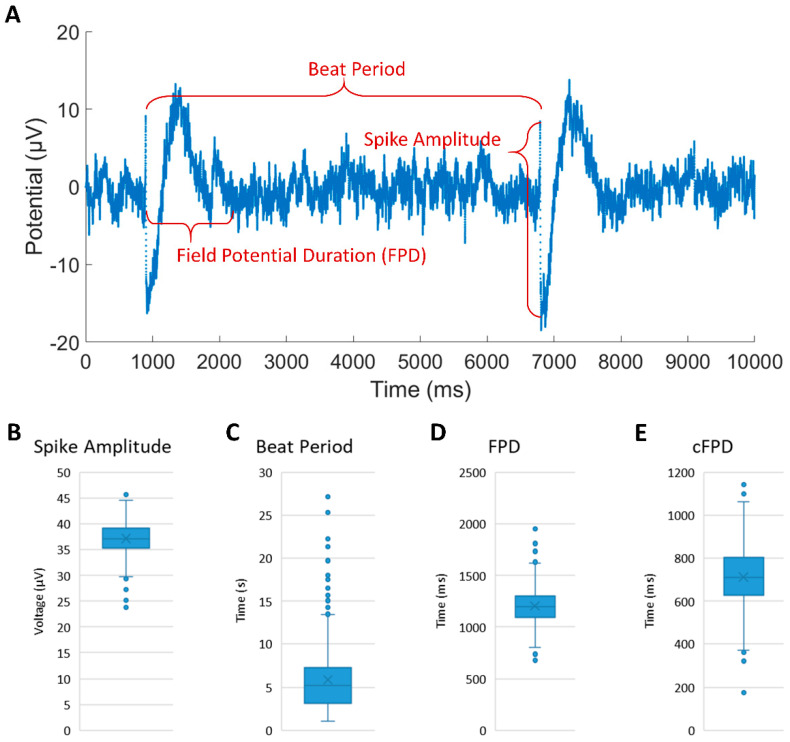
(**A**) The representative electrophysiological parameters and FP waveform of iCM clusters recorded from custom-designed MEA device. (**B**–**E**) Average results of spike amplitude, beat period, field potential duration and corrected field potential duration during 40 min recording.

We assessed the feasibility of monitoring the electrophysiological properties of patterned iCMs using the MEA device. Figure 4A shows the representative FP waveforms of cultured iCMs with an average spike amplitude of 37.12 ± 3.05 μV, a beat period of 5.88 ± 3.87 s, an FPD of 1203.36 ± 172.83 ms and a cFPD of 712.48 ± 141.58 ms (shown in Figure 4B–E) during the 40 min recording. The spike amplitudes were constant during the entire recording duration, while the beat period, FPD and cFPD were slightly dispersed. The spike amplitude values we recorded are smaller than previously reported values [23,24], most likely due to the relatively large cell-electrode distance, which results in attenuation during signal transmission. The results of FPD and cFPD are similar to the literature. Compared with rat cardiomyocytes, iCMs have different electrical properties, such as lower spike amplitude and slower beating rhythm. The extracellular recording of iCMs proved that our customized MEA device can be used to detect other types of cardiomyocytes even with smaller currents. Combining the MEA device with our micropatterning method provides an approach to guide the coupling pathway in the iCM-fibroblast network, which can be further used to study the influence of iCM synchronization on cell maturity by monitoring the long-term electrical activity.

Previous studies have described strategies that combine MEA with micropatterning techniques to detect the electrophysiology of patterned electrically active cells, including neurons and CMs (Table 1). In most cases, their micropatterning methods are achieved by pre-treating the culture surface chemically [13,25], mechanically [26,27] or topographically [12,27,28,29,30], aiming to record the electrical activities of a single CM or anisotropic cardiac cultures. However, there is not much research on controlling the connection between CMs and fibroblasts. Recently, the Cazorla group and Pimashkin group [31,32] published micropatterning methods that guided the growth direction of axons using microchannels to connect neuron networks unidirectionally, achieving organized neuronal networks. Here, we applied a similar micropatterning method with surface topographic features and mobile blockers to control the cardiac cells’ localization and the connections in the rCM-fibroblast network by obstructing the initial cell attachment and subsequently allowing cell proliferation in permissive areas. The coupling dynamics in such controlled rCM-fibroblast networks was investigated through the long-term and non-invasive electrical monitoring on rCM clusters and the locations of interest in the custom-designed MEA device. Our results indicate that the fibroblast bridges allow the electrical coupling between physically separated rCM clusters by propagating the electrical signals generated by rCM clusters. The synchronized rCM clusters also display stable phase differences, which are likely caused by the time lag in propagating the electrical signals along the bridges. Recent studies have suggested that such propagation delay may cause conduction disturbances and eventually lead to arrhythmogenesis [2]. Our micropatterning method, favorable for creating controlled connections between rCMs and fibroblasts, has the potential to obtain a more complex cardiac cell network to better mimic normal or diseased cardiac tissue.

## 4. Summary and Conclusions

In conclusion, this paper introduces a novel micropatterning method for defining a controlled network of CM and fibroblast connections on a custom-designed MEA device. The surface topographical features and mobile blockers were utilized to pattern the neonatal rat cardiac cells, rCMs and fibroblasts into separate rCM clusters and fibroblast bridges by obstructing the initial cell attachment and subsequently allowing fibroblast growth. The proliferated fibroblasts would connect and hence couple the separate rCM clusters. The coupling dynamics among the rCM-fibroblast network were recorded by the customized MEA device, which was fabricated by standard photolithography, metal evaporation and the lift-off process. The electrode layout of the MEA device was designed to match the cardiac cell patterning, allowing the measurement at specific locations. Immunofluorescent staining was utilized to validate the cell patterning by showing rCM and fibroblast markers, Troponin T and Vimentin, in the cluster area, while only fibroblast markers were shown in the pre-reserved bridge area. The coupling between the three separate rCM clusters was proved by the same beating frequency in waveforms and calcium flux videos. Stable phase differences between coupled rCM clusters were also observed, which were caused by the time lag in transporting electrical signals via fibroblast bridges among adjacent clusters. The impedance profile of the fibroblast connection was recorded from the sensor with same fabrication process, indicating that the fibroblast bridge could be presented as an RC filter of 200 kΩ resistance and 120 nF capacitance and provided an RC-type coupling among separate rCM clusters. The MEA also showed its efficacy for monitoring the FP of iCMs. Our micropatterning method makes a complex and tunable CM-fibroblast network feasible, which may have further applications in studying the synchronization mechanism in cardiac tissue.

## Figures and Tables

**Table 1:** A brief summary of current technologies of customized MEA for CMs.

Description	Cell Source	Patterns Description	MEA Platform and Electrode Materials	Electrode Arrangement	Ref.
Study the activity-dependent intracellular dynamics in reconstituted neuron networks	Neuron	Single directed axon isolated by microfluidic device	Pt electrode on custom MEA	In the target chambers	Moutaux et al. [31]
Propose a design of organized neuron network with directed connectivity	Neuron	Unidirectional axon growth in microchambers with various shapes	Ti electrode on commercial MEA	In segments of microchambers	Gladkov et al. [32]
Design neuronal circuits with various topologies	Neuron	Neuron bodies in microwell and neurites in microgroove	TiN electrode on commercial MEA	In microwell	Joo et al. [28]
Describe a microfluidic device to measure FP from a single CM	CM	Single CM trapped in microfluidic device	Pt electrode on custom MEA	In microchambers	Werdich et al. [26]
Describe a microchamber to isolate single CM with electrical stimulation in small volumes	CM	Single isolated CM cell in each microchannel	Au electrode on custom MEA	Stimulation and reference electrodes in each microchamber	Klauke et al. [27]
Enable measurement of extracellular FP of a single CM	CM	Single CM individually seeded in hollow microchambers	ITO electrode on custom MEA	In each microchamber	Kaneko et al. [12]
Enable measurement of conductivity, viscosity and refractory period of CMs	CM	A monolayer CM with long path on the surface modified by PEG and fibronectin	Ti electrode on commercial MEA	In the start and end positions of path	Natarajan et al. [25]
Develop multi-well MEA plates with nanotopographic patterns	CM /Neuron	Aligned cells along the topographic nanogrooves	Custom MEA	Electrode matrix in each well	Smith et al. [29]
Study the electrical activity of iCMs on aligned fibers	CM	Anisotropic iCMs on the surface modified by electrospun aligned fiber	Ti electrode on commercial MEA	/	Li et al. [30]
Investigate the physical and protein cues to engineer aligned iCMs	CM	Anisotropic monolayer of iCMs along the direction of printed proteins and microgrooves	Ti electrode on commercial MEA	/	Alassaf et al. [13]
Study the coupling dynamics of a controlled network of rCM and fibroblast connections	CM /Fibroblast	Three individual rCM clusters and connected fibroblast bridges patterned by topographic features and mobile blockers	Au electrode on custom MEA	In CM clusters and fibroblast connection pathway	This paper

## Data Availability

The raw/processed data required to reproduce these findings can be shared upon reasonable request.

## Supplementary Materials

The following are available online at https://www.mdpi.com/article/10.3390/mi12111351/s1, Video S1: Supplementary Movie 1.
